# Serum antinucleosome-specific antibody as a marker of autoimmunity in children with autism

**DOI:** 10.1186/1742-2094-11-69

**Published:** 2014-04-03

**Authors:** Laila Yousef AL-Ayadhi, Gehan Ahmed Mostafa

**Affiliations:** 1 Autism Research and Treatment Center, AL-Amodi Autism Research Chair, Department of Physiology, Faculty of Medicine, King Saud University, Riyadh, Saudi Arabia; 2 Department of Pediatrics, Faculty of Medicine, Ain Shams University, 9 Ahmed El-Samman Street off Makram Ebaid, Nasr City 11511 Cairo, Egypt

**Keywords:** Antinucleosome-specific antibodies, Autism, Autoimmunity, Family history of autoimmunity

## Abstract

**Background:**

Increasing evidence of autoimmune phenomena in some individuals with autism could represent the presence of altered or inappropriate immune responses in this disorder. The role of the nucleosome in the induction of antibody response in some autoimmune-mediated tissue damage may provide novel targets for treatment. Due to the paucity of studies investigating the frequency of systemic auto-antibodies in autism, we are the first to investigate the frequency of antinucleosome-specific antibodies in a group of autistic children.

**Methods:**

Serum antinucleosome-specific antibodies were measured by ELISA in 60 autistic children, between the ages of 3 and 12 years, in comparison to 60 healthy children. Autistic severity was assessed using the Childhood Autism Rating Scale (CARS).

**Results:**

Autistic children had significantly higher serum antinucleosome-specific antibodies than healthy children (*P* <0.001). The seropositivity of antinucleosome-specific antibodies was found in 46.7% of autistic children. Autistic children with a family history of autoimmunity (40%) had a significantly higher frequency of serum antinucleosome-specific antibodies (83.3%) than patients without such a history (22.2%, *P* <0.001).

**Conclusions:**

Serum levels of antinucleosome-specific antibodies were increased in some autistic children. However, these data should be treated with caution until further investigations are performed with a larger subject population to determine whether these antibodies have a role in the induction of autoimmunity in a subgroup of autistic children. The role of immunotherapy in children with autism should be also studied.

## Background

A possible role of immune system abnormalities in the pathogenesis of some neurologic disorders, including autism, was postulated. Autoimmunity to the central nervous system is the most common of these abnormalities [1,2]. Brain-specific auto-antibodies were detected in the sera of many autistic children [3-10]. In addition, autoimmune disorders are increased in families of some children with autism [11-14]. There is a strong association between autism and the major histocompatibility complex for the null allele of C4B in the class III region. This results in low production of C4B protein, leading to repeated infections that play an important role in the development of autoimmunity [15-18]. Some autistic children have an imbalance of T helper (Th)1/Th2 subsets toward Th2, which are responsible for allergic response and production of autoantibodies [1].

One of the common serological hallmarks of autoimmune disorders is the presence of various autoantibodies in the sera of patients affected by these disorders [19]. The presence of abnormal levels of autoantibodies to intracellular antigens is a hallmark of several autoimmune diseases [20].

Evidence accumulated in recent years suggests that the nucleosome, the fundamental unit of chromatin and a normal product of cell apoptosis, plays a key role in some autoimmune diseases as it is a major target autoantigen for autoantibody mediated tissue lesions [21,22]. The broad antinucleosome antibody family includes: the nucleosome-specific antibodies (antinucleosome antibodies without anti ds-DNA and antihistone reactivities), the antinucleosome antibodies with anti ds-DNA reactivity, and the antinucleosome antibodies with antihistone reactivity [23]. Anti ds-DNA antibodies account for a minor part (<30%) of the serum antinucleosome reactivity in lupus patients and nucleosome-specific autoantibodies are in large excess over anti ds-DNA in lupus patients [24].

Due to the paucity of studies investigating the frequency of systemic auto-antibodies in autism, we are the first to investigate the frequency of antinucleosome-specific antibodies in a group of autistic children.

## Methods

### Study population

This case-control study was conducted on 60 children who had classic-onset autism, over a period of 6 months from the beginning of June 2013 to the end of November 2013. The autistic group comprised 49 male and 11 female patients recruited from the Autism Research and Treatment Center, Faculty of Medicine, King Saud University, Riyadh, Saudi Arabia. Patients fulfilled the criteria for the diagnosis of autism according to the 4th edition of the Diagnostic and Statistical Manual of Mental Disorders [25]. Their ages ranged from 3 to 12 years (median (IQR) = 7 (3) years). Patients who had associated neurological diseases (such as cerebral palsy and tuberous sclerosis) or metabolic disorders (such as phenylketonuria) were excluded from the study.

The control group comprised 60 age- and sex-matched apparently healthy children. They included 50 male and 10 female children. They were the healthy older siblings of the healthy infants who attend the Well Baby Clinic, King Khalid University Hospital, Faculty of Medicine, King Saud University, Riyadh, Saudi Arabia for routine follow-up of their growth parameters. The control children were not related to the children with autism and demonstrated no clinical findings suggestive of immunological or neuropsychiatric disorders. Their ages ranged from 3 to 12 years (median (IQR) = 6 (4) years).

This study was approved by the local Ethical Committee of the Faculty of Medicine, King Saud University, Riyadh, Saudi Arabia. In addition, an informed written consent of participation in the study was signed by the parents or the legal guardians of the all studied subjects.

### Study measurements

#### Clinical evaluation of autistic patients

This was based on clinical history taking from caregivers, clinical examination and neuropsychiatric assessment. In addition, assessment of the disease severity was done using the Childhood Autism Rating Scale (CARS) [26], which rates the child on a scale from one to four in each of fifteen areas (relating to people; emotional response; imitation; body use; object use; listening response; fear or nervousness; verbal communication; non-verbal communication; activity level; level and consistency of intellectual response; adaptation to change; visual response; taste, smell and touch response; and general impressions). According to this scale, children who have scored 30 to 36 have mild to moderate autism (n = 31), while those with scores ranging from 37 to 60 points have severe autism (n = 29).

In addition, a family history of autoimmune diseases in controls and children with autism was ascertained in an identical fashion, but not in a blinded manner, by an expert rheumatologist. Parents were asked to fill out a questionnaire regarding which first- (parents and sibs) or second-degree relatives (grandparents, uncles and aunts) had received a diagnosis of specified autoimmune disorders. A list of autoimmune diseases with descriptions was provided. There was a verification of the diagnosis of autoimmune diseases via medical record review. The disorders inquired about in the questionnaire included rheumatoid arthritis, juvenile rheumatoid arthritis, systemic lupus erythematosus, insulin-dependent diabetes mellitus, rheumatic fever, vasculitis, ankylosing spondylitis, dermatomyositis, polymyositis, scleroderma, uveitis, Sjogren’s syndrome, polyarteritis nodosa, Wegener’s granulomatosus, Takayasu’s arteritis, psoriasis, multiple sclerosis, vitiligo, myasthenia gravis, amyotrophic lateral sclerosis, Crohn’s disease, ulcerative colitis, autoimmune thyroiditis, idiopathic thrombocytopenic purpura, Addison’s disease, pemphigus, and Guillain-Barre syndrome. These disorders were chosen because all have a known or suspected autoimmune cause.

#### Measurement of antinucleosome-specific antibodies

This was performed by the ELISA technique (EUROIMMUN Medizinische Labordiagnostika AG, Germany) [27]. The samples were run randomly in a blinded fashion and they were run together, in parallel on the same run with the same internal standards. Antibodies to highly purified human nucleosomes (antinucleosome-specific antibodies), if present in diluted serum, would bind in the microwells. Washing the microwells removes unbound serum antibodies. Horseradish peroxidase conjugated anti-human IgG immunologically binds to the bound patient antibodies, forming a conjugate/antibody/antigen complex. When substrate is washed in the presence of bound conjugate, it is hydrolyzed to form a blue color. The addition of an acid stops the reaction, resulting in a yellow end product. The intensity of this yellow color is measured photometrically at 450 nm. The amount of color is directly proportional to the concentration of IgG antibodies present in the original sample. To increase accuracy, all samples were analyzed twice in two independent experiments to assess interassay variations and to ensure reproducibility of the observed results (*P* >0.05). No significant cross-reactivity or interference was observed.

### Statistical analysis

The results were analyzed by a commercially available software package (Statview; Abacus Concepts, Inc., Berkley, CA, USA). The data were non-parametric; thus, they were presented as median and interquartile range (IQR), which are between the 25th and 75th percentiles. A Mann- Whitney test was used for comparison between these data. A chi-square test was used for comparison between qualitative variables of the studied groups. A Spearman’s rank correlation coefficient ‘r’ was used to determine the relationship between different variables. For all tests, a probability (*P*) of less than 0.05 was considered significant. As data distribution was non-parametric, serum antinucleosome-specific antibodies levels were considered to be elevated if there levels were above the 95th percentile of the healthy control values (3.49 U/l).

## Results

The characteristics of the study subjects are shown in Table 1.

**Table 1:** Demographic data of children with autism and healthy controls

	**Children with autism (n = 60)**	**Control group (n = 60)**
Age (in years)	7 (3)	6 (4)
Sex (male/female)	49/11	50/10
Family history of autoimmune diseases	24/60 (40%)	5/60 (8.3%)
Rheumatoid arthritis	14	3
Insulin-dependent diabetes mellitus	4	1
Systemic lupus erythematosus	3	1
Autoimmune thyroiditis	2	
Rheumatic fever	1	

### Serum levels of antinucleosome-specific antibodies in healthy children and patients with autism

Autistic children had significantly higher serum levels of antinucleosome-specific antibodies than healthy controls, *P* <0.001 (Table 2). According to the highest cut-off value of serum antinucleosome-specific antibodies, increased serum levels of antinucleosome-specific antibodies were found in 46.7% (28/60) of autistic children.

**Table 2:** Serum levels of antinucleosome-specific antibodies in autistic patients and healthy children

	**Serum antinucleosome-specific antibodies**	**Z**
	**Median (IQR)**	**(*P*)**
Healthy children (n = 60)	1.7 (1)	5.6
Autistic patients (n = 60)	2.9 (2.6)	(<0.001)
Mild to moderate autism (n = 31)	3.4 (3)	0.4
Severe autism (n = 29)	2.8 (2.7)	(0.62)

IQR, interquartile range.

On the other hand, there was a nonsignificant difference between serum levels of antinucleosome-specific antibodies of children with mild to moderate autism and patients with severe autism, *P* = 0.62 (Table 2). Also, male and female patients with autism had no significant statistical difference between them in serum levels of antinucleosome-specific antibodies (*P* = 0.32). Serum levels of antinucleosome-specific antibodies had significant correlations neither with the age of autistic children (*P* = 0.48) nor with CARS (*P* = 0.50).

### A family history of autoimmune diseases in patients with autism and its relation to the frequency of antinucleosome-specific antibodies

Twenty-four children with autism (40%) had a first- or a second-degree relative with an autoimmune disease (rheumatoid arthritis in 14 patients, insulin-dependent diabetes mellitus in 4 patients, systemic lupus erythematosus (SLE) in 3 patients, autoimmune thyroiditis in 2 patients and rheumatic fever in 1 patient), Table 1. Six out of the 24 autistic children with a family history of autoimmune disease (25%) had a mother with an autoimmune disease (three had rheumatoid arthritis, one had SLE, one had insulin-dependent diabetes mellitus and one had autoimmune thyroiditis).

On the other hand, a family history of autoimmune diseases was found in 5 of the studied 60 (8.3%) healthy children (rheumatoid arthritis in 3 children, SLE in 1 child and insulin-dependent diabetes mellitus in 1 child), Table 1. None of the healthy children with a family history of autoimmune disease had a mother with such diseases. The frequency of autoimmune diseases among families of children with autism was significantly higher than normal children (*P* <0.001).

Autistic children with a family history of autoimmunity had significantly higher frequency of serum antinucleosome-specific antibodies (83.3%) than patients without such a history, (22.2%, *P* <0.001) (Table 3).

**Table 3:** The frequency of antinucleosome-specific antibodies in relation to a family history of autoimmune diseases in autistic patients

**Children with autism (n = 60)**	**Antinucleosome-specific antibodies**		**X**^**2 **^**(*P*)**
	**Seropositive (n = 28)**	**Seronegative (n = 32)**	
A positive family history of autoimmune diseases (n = 24)	20 (83.3%)	4 (16.7%)	21.6
No family history of autoimmune diseases (n = 36)	8 (22.2%)	28 (77.8%)	(<0.001)

## Discussion

Autoimmunity may have a role in the pathogenesis of autism. Immune system dysfunction may represent novel targets for treatment in autism [1-3]. In our series, autistic children had significantly higher serum levels of antinucleosome-specific antibodies than healthy controls (*P* <0.001). According to the highest cut-off value of serum antinucleosome-specific antibodies, increased serum levels of these antibodies were found in 46.7% of autistic children. We could not trace data in literature regarding the frequency of antinucleosome-specific antibodies in children with autism to compare with our results.

Pathological T cell clones that recognize double-stranded DNA and nucleosomes further drive B cell production of anti-DNA and antinucleosome autoantibodies. Deposition of these autoantibodies within the brain and other organ systems contributes to the pathophysiology and clinical manifestations of autoimmune diseases such as SLE [28]. Complement-fixing IgG autoantibodies including anti-DNA and antinucleosome antibodies may cross the blood-brain barrier and combine with brain tissue antigens to form immune complexes that damage the neurological tissue in autistic patients [4].

The term ‘nucleosome’ defines a basic unit of chromatin. Each nucleosome consists of 146 base pairs of double stranded DNA, wrapped twice around a histone octamer, a protein core. A histone octamer consists of two molecules each of histones H2A, H2B, H3, and H4. In chromatin, nucleosomes are connected by 15 to 80 base pairs of linker DNA, to which histone H1 is attached. Anti-dsDNA and anti-histone antibodies belong to the nucleosome family as do antinucleosome-specific antibodies, since nucleosomes share several common epitopes with dsDNA and histones. Nucleosome-specific antibodies do not react with the individual components of the nucleosome (that is, DNA and histones) but recognize conformational epitopes resulting from interactions between the DNA and histone [29]. Nucleosomes are generated *in vivo* by the process of apoptosis, which is disturbed in some autoimmune diseases such as SLE. Nucleosomes are the major target autoantigens for autoantibodies mediating tissue lesions, especially glomerulonephritis in SLE [30,31]. Previous studies have reported that antinucleosome antibody reactivity is a very sensitive marker of SLE [32-37]. Similarly, it has been reported that 30% of SLE patients with high antinucleosome-specific antibody reactivity have little, if any, anti ds-DNA or antihistone reactivity [34].

The mechanisms that lead to the induction of antinucleosome specific autoantibodies in some autoimmune diseases remain obscure. In view of the prominence of nucleosomes which circulate at high levels in some autoimmune diseases such as SLE [38], it has been speculated that highly accelerated rates of apoptosis [39], and/or abnormal sites or abnormal processing of apoptotic cells could lead to autoantibody production [40]. Also, nucleosomes may elicit the production of interleukin-6 and stimulation of lymphoproliferation and IgG synthesis by splenic B cells. This could result in a polyclonal activation that triggers both a specific (nucleosome-driven) and nonspecific antibody production [38]. Alteration of the selected parameters confirm the role of apoptosis and neuroinflammation mechanisms in the etiology of autism [41]. The apoptotic marker soluble fatty acid synthase antigen was reported to be high in Saudi children with severe autism, and can be considered an indicator of disease severity [42]. Disturbances in brain glutathione homeostasis may contribute to oxidative stress, immune dysfunction and apoptosis, particularly in the cerebellum and temporal lobe, and may lead to neurodevelopmental abnormalities in autism [43]. Thus, accelerated rates of apoptosis in autism, like in SLE, may be the possible reason behind the increased frequency of antinucleosome-specific autoantibodies in some autistic children as shown in this study. Additional investigation designed to expand on these data is warranted.

Other possible reasons behind the initiation of autoimmunity and the production of autoantibodies in some autistic children may be attributed to the exposure to some environmental cross-reacting antigens, which initiate autoimmune reactions in genetically susceptible individuals [4]. These environmental antigens include food allergies to certain peptides as casein of milk and gluten of wheat [44,45], heavy metals exposure [46,47] and *Hevea brasiliensis* proteins in natural rubber latex [48]. In addition, infectious agents (for example, virus-induced autoimmunity) may play a causal in autism [1,49].

Studies investigating the frequency of systemic antibodies in autism are very few. Seropositivity of antinuclear antibodies was reported in only 20% of 80 Egyptian children with autism, and this percentage was significantly higher than healthy children (2.5%) [7]. Brain specific autoantibodies were reported in sera of a large proportion of children with autism [4-10]. This may be attributable to the imbalance of T helper (Th)1/Th2 subsets toward Th2, which are responsible for the production of antibodies and allergic response, in some children with autism [1]. In 2008, Mostafa and associates [6] reported seropositivity for antimyelin-associated glycoprotein antibodies in 62.5% of a group of 32 Egyptian autistic children between 3 and 8 years of age.

The explanation of the lower percentage of systemic antibodies (such as antinuclear antibodies and antinucleosome-specific antibodies) than the percentage of brain-specific autoantibodies reported by other studies [4-10] may be explained by the fact that autoimmunity in autism is organ-specific (that is, to brain) and not multisystemic. Diseases with multisystem autoimmunity (for example, SLE) have an increased frequency of systemic antibodies (such as antinuclear antibodies and antinucleosome-specific antibodies) and in these diseases, measurement of systemic antibodies is a reliable screening test [14]. Thus, testing for brain-specific autoantibodies seems to be more reliable than an antinucleosome-specific antibodies test in screening for autoimmunity in autism.

To further understand if autoimmunity could play a role in autism, we studied the frequency of autoimmune diseases in families of patients with autism in comparison to healthy children. The frequency of autoimmune disease among families of the former group (40%) was significantly higher than that of the latter group (8.3%). Previous research had also found an increased frequency of autoimmunity in families of children with autism compared to those of healthy and autoimmune control subjects [11-15]. In one study [11], a family history of autoimmune diseases was reported in 46% of children with autism. They also reported that as the number of family members with autoimmune disorders increased from one to three, the risk of autism was greater with an odds ratio that increased from 1.9 to 5.5, respectively. Thus, this may be an outstanding feature among patients with autism that points to their autoimmune background, with the target in this case being the developing brain.

In our series, the finding of the increased frequency of autoimmune diseases in the mothers of children with autism (25%) was also in agreement with that of Comi *et al*. [11] who reported an autoimmune disease in 16% of the mothers of their studied patients with autism. The high rate of autoimmune diseases in the mothers of the children with autism could also suggest that an autoimmune process exists in the mothers that targets the developing fetus *in utero*. Although this would be more consistent with the female preponderance in autoimmune disorders, it does not explain the high male-to-female ratio observed in autism [12].

The current study revealed a more significant increase of the frequency of serum antinucleosome-specific antibodies in autistic children with a family history of autoimmunity (83.3%) than patients without such a history (22.2%), *P* <0.001. This implies that in some families, immune dysfunction, perhaps induced by certain environmental triggers, could express itself in the form of autism in one of its offspring. Immune-related genes in the major histocompatibility complex (MHC) may play a central role in the development of autoimmunity in autism. These genes have been associated with some autoimmune diseases such as systemic lupus erythematous and diabetes mellitus. A previous study reported that mothers and their sons had a significantly higher frequency of HLA-DR4 than normal control subjects [50].

To date, a definitive relationship between autism and autoimmunity has not been fully established. On the basis of the preliminary results reported in this study, however, there seems to be a suggestion of evidence in support of autoimmune contributions to the pathophysiology of autism in some cases. Additional investigation designed to expand on these data is warranted.

## Conclusions

Serum levels of antinucleosome-specific antibodies were increased in some autistic children. However, these data should be treated with caution until further investigations are performed with a larger subject population to determine whether these antibodies have a role in the induction of autoimmunity in a subgroup of autistic children. The role of immunotherapy in children with autism should be also studied.

## Abbreviations

CARS: Childhood Autism Rating Scale; IQR: interquartile range; SLE: systemic lupus erythematosus; Th: T helper cells.

## Competing interests

The authors declare that they have no competing interests.

## Authors’ contributions

Both authors designed, performed and wrote the research. In addition, both authors read and approved the final manuscript.
